# Asymptomatic testing people for SARS-CoV-2 in healthcare facilities: A systematic review

**DOI:** 10.4102/jphia.v16i2.581

**Published:** 2025-01-03

**Authors:** Olabisi A. Oduwole, Glory Bassey, Grace Esebanmen, Samuel Shoyinka, Johnsolomon Ohenhen, Elise Cogo, Nicholas Henschke, Eleanor Ochodo, Martin M. Meremikwu

**Affiliations:** 1 Department of Medical Microbiology and Parasitology, Faculty of Medical Laboratory Science, Achievers University, Owo, Nigeria; 2 Cochrane Nigeria, Institute of Tropical Diseases Research and Prevention, University of Calabar Teaching Hospital, Calabar, Nigeria; 3 Department of Paediatrics, University of Calabar Teaching Hospital, Calabar, Nigeria; 4 Nigeria Centre for Disease Control and Prevention, Abuja, Nigeria; 5 Cochrane Response, London, United Kingdom; 6 Centre for Global Health Research, Kenya Medical Research Institute, Kenya, Kenya

**Keywords:** asymptomatic SARS-CoV-2, COVID-19 test, asymptomatic testing, asymptomatic diagnosis, asymptomatic screening, asymptomatic COVID-19

## Abstract

**Background:**

Asymptomatic testing involves the process whereby individuals who do not show symptoms of COVID-19 are tested for severe acute respiratory syndrome coronavirus 2 (SARS-CoV-2) infection using any of the available laboratory test techniques.

**Aim:**

To evaluate the effectiveness of testing asymptomatic individuals visiting, living or working in healthcare facilities in reducing SARS-CoV-2 viral infections.

**Setting:**

Healthcare databases.

**Method:**

Electronic databases were searched and limited to English language and studies published 2020 to 02 September 2022. Following the methods for rapid systematic reviews, data were analysed using a fixed effect model, and results of the effect estimate were reported as odds ratios (OR) with their confidence intervals (CI) (95% CI).

**Results:**

Databases’ searches yielded 3065 articles after deduplication and 3 studies by searching reference lists of included articles. After screening abstracts and full text articles, 3 cohort studies were included, each with serious risk of bias. Very low certainty evidence shows a decrease in occurrence of SARS-CoV-2 infections in the asymptomatic testing group among patients going for index surgery (OR: 0.05, 95 % CI: 0.00–0.82; 501 participants; 1 study) and among long term care facility staff (OR: 0.31, 95 % CI: 0.18–0.52; 3457 participants; 2 studies, *I*^2^ = 89%) than the ‘no asymptomatic testing’ group. However, its effect on their residents was contradictory.

**Conclusion:**

There is limited quality evidence to support asymptomatic testing of individuals for SARS-CoV-2 in the prevention of virus transmission in health care settings.

**Contribution:**

In the event of a future pandemic, this review offers current evidence on the potential effects of asymptomatic testing.

## Introduction

COVID-19 is a pandemic caused by a virus known as severe acute respiratory syndrome coronavirus 2 (SARS-CoV-2). The COVID-19 pandemic remained persistent because of the ability of the causative agent to mutate into different variants, each variant with varying degree of virulence.^1^ There are effective vaccines for preventing the spread of SARS-CoV-2; however, it is not clear which of the available COVID-19 vaccines are long lasting. Furthermore, the immunity conferred by these vaccines on individuals varies depending on the variant of exposure.^2^ Globally, this disease has had enormous effects on morbidity and mortality, and has led to enormous economic loss.^3^ As at 10 August 2022, according to World Health Organization (WHO) report, over 500 million confirmed COVID-19 cases have been reported worldwide and approximately 6 million people have died from the disease.^4^ However, the WHO recently declared in 2023 that COVID-19 was no longer a pandemic, as evidenced by a global decline in incidence and mortality of approximately 40%.^5^ At the peak of COVID-19 pandemic, healthcare workers (HCWs) were at high risk of exposure to nosocomial transmissions of SARS-CoV-2 and have been heavily impacted by the COVID-19 pandemic, especially at the early stage of the outbreak.^6^ Available evidence shows that SARS-CoV-2 is largely transmitted through person-to-person contact;^7^ therefore, early detection of infection is critical to reducing transmission and screening exposed individuals regardless of symptoms. It is estimated that between 1% and 17% of SARS-CoV-2 infections worldwide are asymptomatic.^8^ Therefore, screening asymptomatic individuals such as visitors and people working in healthcare settings may further reduce the spread of SARS-CoV-2 and help monitor the epidemiology of the disease.^9^

Asymptomatic testing involves the process whereby individuals who do not show symptoms of COVID-19 are tested for SARS-CoV-2 infection using any of the available laboratory test techniques. Severe acute respiratory syndrome coronavirus 2 infections can be detected using nucleic acid amplification tests (NAAT) such as reverse transcriptase-polymerase chain reaction (RT-PCR), multiplex polymerase chain reaction (PCR), and nested PCR. Other screening methods are antigen-based rapid diagnostic test kit (RDT) and antibody-based tests.^10^

### How the intervention might work

Asymptomatic individuals and those in the prodromal stage of SARS-CoV-2 (the period just before signs and symptoms appear in infected cases) could unknowingly transmit the virus to close contacts.^3^ Asymptomatic testing allows early detection of cases and isolation of individuals who are asymptomatic and in the prodromal stage of SARS-CoV-2, thus stopping the spread of the virus.^8,11^

In a healthcare setting or nursing home, asymptomatic testing may be very useful in reducing the spread of SARS-CoV-2 and also preventing adverse outcomes of COVID-19 among HCWs and residents of care homes. In addition, antibody-based testing could be helpful in determining the proportion of population that have been previously exposed either by previous infection or by vaccination.^12^

### Why is it important to do the review

Asymptomatic infections are one of the leading contributors to the transmission of SARS-CoV-2,^13^ and are often missed in active surveillance. Furthermore, asymptomatic SARS-CoV-2 infections may have harmful effects on the lung if not detected early.^14^ It has been advocated that not only symptomatic individuals should be screened for COVID-19 infection, but also asymptomatic individuals, particularly unvaccinated groups, should be screened with available testing methods.^9,10^ This approach is considered key to strategies to prevent transmission of SARS-CoV-2.^8^ The WHO guideline recommends that asymptomatic individuals working in healthcare facilities and nursing homes should be regularly tested for SARS-CoV-2.^15^ This policy has serious cost implications for poor countries as it will amount to billions of dollars to implement successfully.^16^ It is unclear whether testing asymptomatic people in healthcare facilities will reduce SARS-CoV-2 infections in these settings. Thus, this rapid systematic review aims to evaluate the effectiveness of testing asymptomatic individuals visiting or working in healthcare facilities in reducing SARS-CoV-2 viral infections.

### Objective

To assess the effectiveness of COVID-19 tests in screening asymptomatic individuals in reducing SARS-CoV-2 infections in healthcare settings.

### Methods

The review protocol was registered with the International Prospective Register of Systematic Reviews (PROSPERO, CRD42022355749).

### Criteria for considering studies for this review

#### Inclusion criteria

*Types of studies:* Types of studies considered for inclusion were: randomised controlled trials (RCTs) or quasi-RCTs that compared testing to no testing in asymptomatic participants such as in healthcare settings. In the absence of RCTs, we considered the following study types: cohort studies (prospective or retrospective), case-control studies, controlled before-and-after studies (CBA), interrupted time series studies and cross-sectional studies with a control group. We also included studies reporting data on costs of the intervention. We excluded studies reporting only on the diagnostic accuracy of the test intervention, conference abstracts with insufficient information, opinions and non-research editorials:

- *Types of participants:* Studies that included populations with no symptoms of SARS-CoV-2 infection but at risk of infecting or being infected in healthcare settings (e.g., HCWs and other staff; visitors; residents in long term care homes; inpatients; surgical patients; outpatients) were included.
- We excluded studies that exclusively tested participants based on symptoms and previous exposure to a known case of COVID-19.

*Types of interventions:* The intervention considered for this review was asymptomatic testing using any test tool (RT-PCR), antigen-based testing method (RDT, ELISA [enzyme-linked immunosorbent assay]), antibody-based testing method (ELISA, chemiluminescence immunoassay [CLIA], RDT) compared with no testing.

*Types of outcome measures:* Primary outcome measured was number of SARS-CoV-2 infections identified in the facilities; and the secondary outcomes were: (1) cost of testing asymptomatic cases, (2) number of referrals for treatment, (3) time to becoming symptomatic, (4) proportion of contacts infected.

#### Search methods for identification of studies

**FIGURE 1:**
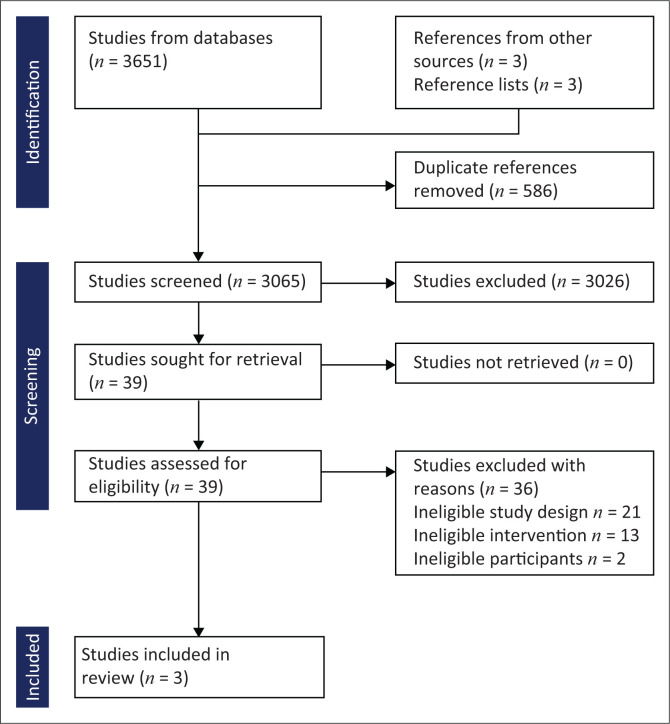
PRISMA flowchart showing selection process.

*Electronic searches:* The following databases were searched by expert information scientists (E.C. in collaboration with other information scientists not involved with the review): the Cochrane Library – Central Register of Controlled Trials (CENTRAL) and Cochrane Database of Systematic Reviews up to 31 August 2022; MEDLINE and EMBASE from the year 2020 to date of the definitive evidence search up to 02 September 2022 (Online Appendix 1). We also searched the reference lists of retrieved full text of relevant studies for additional reports of relevant studies. We restricted search to articles published in English, and used a Preferred Reporting Items for Systematic Reviews and Meta-Analyses (PRISMA) guideline and a flow diagram to report the search and selection of the studies (Figure 1).

### Data collection and analysis

#### Selection of studies

This was a rapid systematic review; as such, one author screened each title and abstract and a second author reviewed for accuracy. Results of the literature search were evenly distributed among co-authors (G.B., E.O., S.S., G.E.) and screened by one author for potentially relevant studies and to obtain the full reports of potentially relevant studies for further assessment. A more experienced author (O.A.O.), randomly by using a computer-generated random numbers, selected 30% of excluded studies for verification. Full texts of the included studies were obtained and two authors independently screened each study for inclusion using the eligibility screening form previously piloted. The studies were scrutinised to ensure each study was included in the review only once. Disagreements were resolved through consensus agreement with the review team. The excluded studies and the reasons for their exclusion are presented in Online Appendix 2.

### Data extraction and management

We used Microsoft Office Excel to develop a piloted data extraction form adapted from the Cochrane data extraction template. Data related to place, race, occupation, gender, religion, education, social capital and socioeconomic status for all included studies were extracted where available. In order to save time, one review author (O.A.O.) extracted data using the data extraction form, and a second author (E.O.) verified for accuracy. Disagreements were resolved through discussion between all review authors.

For each outcome, the following were extracted: the number of participants randomised and analysed in the intervention and control group. Also, the number of events and number of participants per group were obtained. For continuous outcomes, we extracted the numbers assessed in each group along with the arithmetic means and standard deviations for each intervention group. The following data were extracted where reported: (1) study design, study location, study duration, follow-up, mean age, gender and number and type of included participants, exclusion criteria; (2) number tested, number of positive asymptomatic cases at baseline, asymptomatic cases at follow-up, contact infected, time to become symptomatic, number of referral and cost of the intervention; (3) setting and/or level of care (level 1, 2, 3 and 4) and number of participants lost to follow-up.

### Assessment of risk of bias in included studies

One review author (O.O.) assessed the risk of bias (ROB) of each included study using the Cochrane Risk Of Bias In Non-randomized Studies of Interventions (ROBINS-I) tool.^17^ Another author (O.E.) verified all ROB assessment for accuracy. We limited the ROB assessment to the primary outcome. We resolved any disagreements by discussion between review authors. Only observational studies were included in this review; therefore, for cohort studies with a control group, we used the ROBINS-I tool to assess the ROB by determining the likelihood of confounding the effect of the intervention, bias in the study’s participant selection process, bias in the interventions’ classification process, bias resulting from deviations from the intended interventions, bias resulting from missing data, bias in outcome measurement and bias in the choice of the published result. The judgement for each question was ‘yes, probably yes, no, probably no’, and ‘no information’. Depending on the assessment, the overall assessment was rated as low risk, moderate risk, serious risk, critical risk or no information and presented in Table 2.^17^

Furthermore, the balance between comparator groups at baseline with respect to the main confounding factors such as type of test (PCR, antigen, seroprevalence, antibodies), frequency of testing, vaccination status of the participants, ‘natural’ immunity, Ct values for NAAT based tests were assessed. We also considered the methods used by the study authors to mitigate selection bias at the design stage (e.g., matching or restriction to sub-groups) and in their methods of analysis (e.g., the use of stratification or regression modelling), whether all included participants were included in the final analysis and whether study accounted for missing data.

### Measures of treatment effect and data synthesis

This analysis compared ‘asymptomatic testing’ to no testing. The number of participants experiencing the event and the number assessed in each group were presented in a forest plot. In the absence of substantial clinical or methodological heterogeneity, data were pooled in a meta-analysis with odds ratios (ORs) as a measure of effect because they are appropriate effect measures for observational designs.^18^ We used adjusted measures as the primary effect measures when available. This is to control for confounding factors especially from studies with observational designs. Where feasible, the analyses were sub-grouped by population group such as staff and residents of care facilities. Forest plots were used to summarise the findings from included studies. A minimum of two studies were required for meta-analysis; where a meta-analysis was not feasible because of substantial heterogeneity between studies or because of limited included studies, we synthesised the evidence narratively and presented the results as forest plots.

### Assessment of heterogeneity

Where we conducted a meta-analysis, we assessed statistical heterogeneity and clinical heterogeneity because of variation in study design, adjustments for confounding factors and populations using the *I*^2^ statistic. We presented results as forest plots for the following sub-groups: health workers, patients and residents of care homes where there was a substantial heterogeneity (Chi-square test with a *p*-value of 0.10 or *I*^2^ statistic ≥ 50%) and presented the estimate of effect of the intervention using OR with 95% confidence interval (CI) and a fixed-effects model. There was no investigation of publication bias because only few studies contributed to the meta-analysis.

Studies were sorted in the forest plot by study design feature. Data from studies with comparable designs, interventions and outcome measures were combined in meta-analysis. Where the included studies were not sufficiently homogeneous for combination in a meta-analysis, we displayed the results of the studies in a forest plot but suppressed the pooled estimate and presented a narrative summary of the data based on the Synthesis Without Meta-Analysis (SWiM) guidelines.^19^ We used a fixed-effects model based on the consideration of clinical and methodological heterogeneity between studies and tabulated additional evidence where results could not be presented as a forest plot. The offline version of Cochrane Review Manager 5 (REVMAN 5) was used to generate forest plots.

### Assessment of certainty of evidence

The certainty of the evidence was rated for the outcomes reported (number of SARS-CoV-2 infections identified in the facilities; number of referrals for treatment, and time to becoming symptomatic) using the Grading of Recommendations Assessment, Development and Evaluation (GRADE) approach using the software GRADEPRO GDT.^20^ The quality of the evidence for each outcome was assessed using five criteria: study limitations (an assessment of the overall ROB for studies that contributed to the outcome); consistency of effect (an assessment of explained and unexplained heterogeneity); indirectness (an assessment of how directly the included studies address the review question); imprecision (an assessment of the statistical accuracy of the result); and publication bias (an assessment of the risk of publication bias). For observational studies, the certainty of the evidence starts from very low certainty of evidence and could be upgraded for the following reasons: large effect, if there is evidence that the influence of all plausible confounding would reduce a demonstrated effect or suggest a spurious effect when results show no effect and if there is evidence of dose-response gradient. The summary of findings are presented in Table 3.

### Assessment of publication or reporting bias

We did not assess publication bias, because only two studies were included in meta-analysis.

### Ethical considerations

This article followed all ethical standards for research without direct contact with human or animal subjects.

## Results

### Search results

The initial search returned 3651 articles and additional 3 studies by searching the reference lists of included full text articles. After removing duplicates, we screened the titles and abstracts of 3065 records for eligibility and resulted in 39 selected articles, of which 36 studies were removed after full text review with exclusion reasons and study characteristics provided in Online Appendix 2. After assessing the full text articles, we included three studies, two prospective and one retrospective cohort study. This is shown in the PRISMA flow diagram (Figure 1).

### Included studies

Two of the observational studies were carried out in United States (US) (6172 participants),^21,22^ and one^23^ in Germany (1897 asymptomatic and 106 symptomatic participants); we excluded data for symptomatic participants from the analysis. The characteristics of the included studies are presented in Table 1.

**TABLE 1:** Characteristics of included studies.

Study ID	Study design	Country	Sample size	Population	Setting	Mean age ± s.d.	Study duration	Follow- up	Intervention(s)	Comparison(s)	Outcome(s) reported
Axiotakis 2021^19^	Retrospective cohort study	US	501	SARS-COV-2 negative patients undergoing index surgeries	Acute care hospital	56.6 ± 16.9	15 March 2020 and 15 May 2020.	14 days	Routine testing (Pre-operative testing for the SARS-COV-2 virus using PCR assay. ‘Defined as preoperative test as any test within 3 days of the index surgery’)	No pre-operative testing	COVID-19 within 2–14 days post-operatively was determined
Telford 2020^20^	Prospective cohort study	US	5671	Residents and staff of 28 long term care facilities	Long term care facilities	32.7 ± 6.8	31 March 2020 – 18 May 2020	4 weeks	Regular asymptomatic testing in 13 facilities (‘the preventive group’.) Outcome assessment: Collection 1-day mass Testing for consenting staff and residents plus 4 weeks symptoms-based follow-up	Cause-related testing in 15 facilities (‘the response group’.) Outcome assessment: 1-day mass Testing for consenting staff and residents plus 4 weeks symptoms-based follow-up	Baseline infection prevalence, and to identify subsequent cases
Stemler 2022^21^	Prospective Cohort	Germany	1482	HCWs and visitors	Nursing homes	unclear	Early October 2020 to mid-December 2020	unclear	A standard procedure plus voluntary (non-mandatory) testing of visitors and staff for SARS-CoV-2 (regular, 2–3-weekly voluntary PCR testing of HCWs and visitors in INH)	A standard procedure without testing of visitors and employees (No regular testing in CNH) Residents were not tested routinely within this study	Incidence of symptomatic SARS-CoV-2 infection among resident was the primary endpoint; secondary endpoints proportion of asymptomatic and symptomatic SARS-CoV-2 infection among visitors and HCW in INH

US, United States; s.d., standard deviation; SARS-COV-2, severe acute respiratory syndrome coronavirus 2; PCR, polymerase chain reaction; HCWs, healthcare workers; NH, nursing homes; INH, interventional nursing homes; CNH, control nursing homes.

Axiotakis 2021, a retrospective study^21^ (*n* = 501), reported on asymptomatic patients undergoing index surgeries at an acute care hospital. Mean age ± standard deviation (s.d.) was 56.6 ± 16.9, and proportion of women was 54%. This study^21^ compared patients that had pre-operative asymptomatic testing to patients that did not have pre-operative test and followed them up for up to 14 days post-operatively to determine the risk of developing COVID-19 symptoms. Pre-operative test was defined as any test within 3 days of the index surgery.

Telford 2020^22^ was a prospective cohort study conducted at long term care facilities (LTCF) in the US. It involved 5671 participants from 28 LTCFs, including 2868 (50.6%) residents and 2803 (49.4%) staff members (mean staff age ± s.d. was 32.7 ± 6.8 years). The study^22^ compared regular asymptomatic testing in 13 facilities (‘the preventive group’) to cause-related testing in 15 facilities that conducted tests only after there was a confirmed COVID-19 case (‘the response group’). The investigators carried out a 1-day mass testing for consenting staff and residents plus 4 weeks symptoms-based follow-up in the 28 LTCFs to measure the effectiveness of asymptomatic testing.

Stemler 2022, the third included study,^23^ was a prospective cohort study conducted at four long term care nursing homes in Germany. The study compared two facilities that operated standard procedure with additional surveillance by voluntary testing of visitors and staff for SARS-CoV-2 (regular, 2–3-weekly voluntary PCR testing of HCWs and visitors in interventional nursing homes [INH]) to two facilities that operated a standard procedure without routine testing of visitors and employees (no regular testing in control nursing homes [CNH]), but carrying out ad-hoc testing of staff and residents based on symptoms, suspicion or outbreaks (cause-related testing). Asymptomatic and symptomatic staff and visitors were tested (only data for asymptomatic cases are presented in this review). Of note, Stemler 2022^23^ did not test asymptomatic residents in the intervention homes because they were not given approval to do so. However, residents were tested by local health authority when residents show symptoms compatible with COVID-19; thus, data for the residents in the intervention group and control arm of their study were obtained from the records provided by the local health authority. The mean age and ratio of females to males were not reported. Reverse transcriptase-polymerase chain reaction testing was used in all studies.

### Overall risk of bias assessment

The overall ROB of the included studies was serious. The overall ROB of the studies^21,22,23^ was rated as serious because of confounding of the effect of intervention. This may have led to underestimation of cases in the study (Table 2).

**TABLE 2:** Risk of bias assessment of included studies using Cochrane Risk of Bias In Non-randomized Studies of Interventions tool.

S/n	Study ID	Bias because of confounding†	Bias in selection of participants into the study	Bias in classification of interventions	Bias because of deviations from intended interventions	Bias because ofmissing data?	Bias in measurement of outcomes	Bias in selection of the reported result	Overall bias
1	Axiotakis 2021^19^	Serious	Serious risk	Low risk	Low risk	Low risk	Serious risk	Low risk	Serious risk‡
	Support for judgement	Authors reviewed electronic medical records of adult patients who underwent surgery between 15 March 2020 and 15 May 2020. During this period, surgeries were limited to emergent or urgent cases. However, it was not stated whether they adjusted for confounders in the analysis.	They included participants that had preoperative test (any test within 3 days of the index surgery) and excluded patients with earlier test-confirmed COVID-19 infection.	Data obtained from electronic medical records of participants	Probably no. Data were obtained retrospectively from the participants’ record	All participants were accounted for	Some asymptomatic participants were not tested because of emergency.	Not suspected	
2	Telford 2020^20^	Serious risk	Low risk	Moderate risk	Moderate risk	Low risk	Moderate risk	Low risk	serious risk‡
	Support for judgement	It is not clear if there are social economic differences between groups. It is plausible that the control facilities were doing cause-related testing because of funding. It was not stated whether they adjusted for confounders in the analysis.	Only one long term care facility declined testing for all staff members.	New cases were identified using symptoms-based follow-up. This is subjective	It is possible that the intervention/control groups increased the frequency of testing than usual which could lead to overestimation of the effect.	All facilities included were accounted for in the final analysis.	The baseline assessors were not part of the study; however, follow-up assessors were not well described. Also, outcome data for the control facilities and the residents of the intervention facilities were obtained from the local health authority.	Selective reporting Not suspected	
3	Stemler 2022^21^	Serious risk	Low risk	Serious risk	Low risk	Low risk	Serious risk	Low risk	Serious risk‡
	Support for judgement	Baseline incidence of COVID-19 in the facilities were unknown. Also, authors did not control for any post-intervention variables.	INH and CNH were group-matched according to number of residents and facility size.	Data of incidence of COVID-19 infection among residents was obtained from the local authorities. This may not be a true reflection of the effect of the intervention. Also, testing was voluntary during follow-up in the intervention group.	Probably no. Not suspected.	All participants were accounted for.	Although testing was regular, it was voluntary. It is plausible that some participants volunteered to be tested because they had symptoms. This may have resulted in underestimation of the effect estimate.	Reported results were as listed in the study protocol	

S/n, serial number; ID, identification; ROB, risk of bias; INH, interventional nursing homes; CNH, control nursing homes; COVID-19, coronavirus disease 2019.

† , Confounding factors common to included studies: possible contact with COVID-19 patients, level of care facility, social economic status, recall bias;

‡ , ROB assessment: Serious risk.

## Findings

### Effects of interventions

#### Primary outcome

Number of SARS-CoV-2 infections identified.

All three included studies reported on this outcome.

**FIGURE 2:**
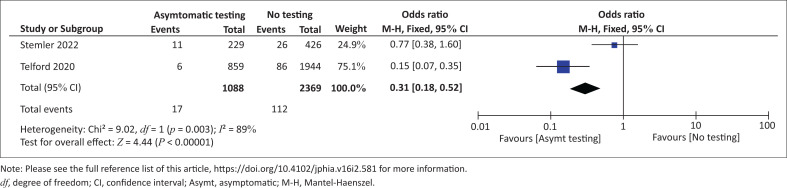
Effect of asymptomatic testing versus no asymptomatic testing on number of severe acute respiratory syndrome coronavirus 2 infections among staff of long term care facilities.

We pooled data for the two studies^22,23^ comparing effects of asymptomatic testing of staff in LTCFs. There was reduced occurrence of SARS-CoV-2 infections among staff of LTCFs that regularly do asymptomatic testing compared to control facilities where testing was based on suspicion or confirmed positive case (OR: 0.31, 95% CI: 0.18–0.52; 3457 participants; two studies; *I*^2^ = 89%, very low certainty evidence; see Figure 2). Because there was substantial heterogeneity in this analysis, we downgraded the certainty of the evidence for very serious inconsistency. The summary of findings is presented in Table 3.

**TABLE 3:** Summary of findings: Asymptomatic testing people for SARS-CoV-2 compared to no testing in healthcare settings.

Outcomes	Anticipated absolute effects†			Relative effect		Participants		Certainty of the evidence (GRADE)	Comments
	Risk with no testing	Risk with asymptomatic testing people	95% CI	OR	95% CI	Number	Observational studies		
Number of SARS-CoV-2 infections at Long term care homes – Number of staff infected	47 per 1000	**15 per** 1000	9–25	0.31	0.18–0.52	3452	2	⨁◯◯◯ Very low^20,21^‡	-
Number of SARS-CoV-2 infection at Long term care homes – Number of residents infected	144 per 1000	10 per 1000	5–17	0.06	0.03–0.10	2868	1	⨁◯◯◯ Very low^20^§	-
Number of SARS-CoV-2 infection at Long term care homes – Number of residents infected	77 per 1000	292 per 1000	196–412	4.98	2.93–8.44	521	1	⨁◯◯◯ Very low^21^¶	-
Number of referrals for treatments – Number of residents referred for treatments	84 per 1000	5 per 1000	2–10	0.05	0.02–0.11	2868	1	⨁◯◯◯ Very low^20^§	-
Number of referrals for treatments – Number of staff referred for treatments	7 per 1000	1 per 1000	0–9	0.16	0.02–1.22	2803	1	⨁◯◯◯ Very low^20^§	-
Number of SARS-CoV-2 infection at a healthcare facility – Number of patients infected	37 per 1000	2 per 1000	0–31	0.05	0.00–0.82	501	1	⨁◯◯◯ Very low^19^¶	-
Time to becoming symptomatic (days)assessed with: daysfollow-up: range 2 days to 28 weeks	-	-	-	‡‡	‡‡	501	1	⨁◯◯◯ Very low^19^††	-
Cost of testing asymptomatic cases – not measured	-	-	-	-	-	-	-	-	-
Proportion of contact infected – not reported	-	-	-	-	-	-	-	-	-

Note: Patient or population: COVID-19; Setting: Healthcare/Long-term care facility settings; Intervention: Symptomatic testing people; Comparison: No testing. GRADE Working Group grades of evidence: High certainty: We are very confident that the true effect lies close to that of the estimate of the effect. Moderate certainty: We are moderately confident in the effect estimate: The true effect is likely to be close to the estimate of the effect, but there is a possibility that it is substantially different. Low certainty: Our confidence in the effect estimate is limited: The true effect may be substantially different from the estimate of the effect. Very low certainty: We have very little confidence in the effect estimate: The true effect is likely to be substantially different from the estimate of effect.

CI, confidence interval; OR, odds ratio; SARS-CoV-2, severe acute respiratory syndrome coronavirus 2; COVID-19, coronavirus disease 2019; GRADE, Grading of Recommendations Assessment, Development and Evaluation.

† , The risk in the intervention group (and its 95% CI) is based on the assumed risk in the comparison group and the *relative effect* of the intervention (and its 95% CI);

‡ , We downgraded by three for a very serious risk of bias because of study limitations, serious indirectness; this was an observational study where participants were not directly randomised to interventions and very serious inconsistency because of a substantial heterogeneity of above 80%;

§ , We downgraded by three for a very serious risk of bias because of study limitations, and serious indirectness. The data was from an observational study in which participants were not directly randomised to interventions;

¶ , We downgraded by three for a very serious risk of bias because of study limitations, and serious indirectness where participants were not directly randomised to interventions and for serious imprecision because of a wide CI;

†† , We downgraded by three for a very serious risk of bias because of study limitations and very serious imprecision because of a wide confidence interval;

‡‡ , 2–10 days.

**FIGURE 3:**
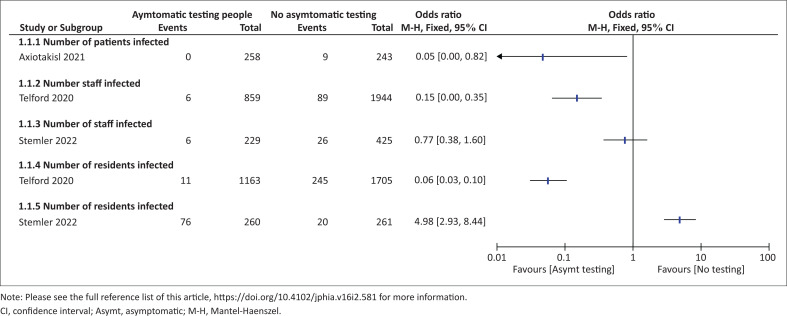
Sub-group analysis of the effect of asymptomatic testing versus no asymptomatic testing on number of severe acute respiratory syndrome coronavirus 2 infections in healthcare facilities.

One study^21^ reported the effect of asymptomatic testing patients before surgery to no testing. The results suggest that there was a decreased occurrence of SARS-CoV-2 infections in the asymptomatic testing group than the ‘no testing’ group (OR: 0.05, 95% CI: 0.00–0.82; 501 participants; one study, very low certainty evidence; see Figure 3). We thus downgraded by two for indirectness and imprecision because of wide CI.

Telford 2020^22^ was carried out in 28 LTCFs (13 intervention and 15 control facilities). This study was rated as having a moderate ROB; as a result, the certainty of the evidence was downgraded for serious study limitation. Very low certainty evidence shows that there may be fewer occurrence of SARS-CoV-2 infection among staff of the asymptomatic testing facilities than the control facilities (OR: 0.15, 95% CI: 0.07–0.35; 2803 participants; one study). Also, the observations from this study suggest fewer occurrence of SARS-CoV-2 infections among residents living in the intervention facilities than the residents of the control facilities (OR: 0.06, 95% CI: 0.03–0.10; 2868 participants; one study, very low certainty evidence; see Figure 3). Stemler 2022^23^ study carried out in four long term care nursing homes (2 INH and 2 CNH) provides very low certainty evidence that there was no difference in the effect of asymptomatic testing people in reducing number of SARS-CoV-2 infections compared to no routine testing in nursing homes. The study^21^ shows that occurrence of SARS-CoV-2 infections was lower among staff of the asymptomatic testing LTCFs compared to the control facilities, although the difference was not statistically significant (OR: 0.77, 95% CI: 0.38–1.60; 654 participants; one study, very low certainty evidence). The certainty of the evidence was downgraded for very serious study limitation, serious imprecision and indirectness of the evidence. In contrast, very low certainty evidence showed there was a higher occurrence of SARS-CoV-2 infection among the residents of the asymptomatic testing facilities (where staff and visitors were tested) than the control group (OR: 4.98, 95% CI: 2.93–8.44; 521 participants; one study). Furthermore, Stemler 2022^23^ reported that 3 out of 722 visitors (0.41%) had SARS-CoV-2 infection in interventional LTCFs, while no information was available for the control facilities (Figure 3).

#### Secondary outcomes

**FIGURE 4:**
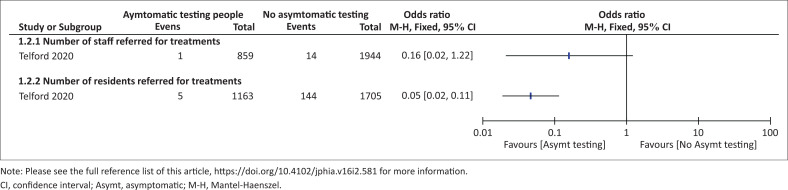
Effect of asymptomatic testing versus no asymptomatic testing on number of participants referred for treatment in long term care facilities.

One study^22^ reported number of participants referred for treatment. The study showed that fewer staff (OR: 0.16, 95% CI: 0.02–1.22; 2803 participants; very low certainty evidence) and residents (OR: 0.05, 95% CI: 0.02–0.11; 2868 participants, low certainty evidence) in the asymptomatic testing group were referred for treatment compared to the control facilities (Figure 4).

Axiotakis 2021^21^ reported that the time to become symptomatic for COVID-19 by participants in the control group (no asymptomatic testing) varied from 2 days to 10 days (very low certainty evidence).

None of the studies reported other outcomes such as cost of the intervention and proportion of contacts infected.

## Discussion

### Summary of main results

This rapid review aimed to determine whether testing people who are asymptomatic for COVID-19 will reduce transmission of SARS-CoV-2 infections in healthcare settings. It included three cohort studies from the US and Germany, two of which were pooled in a meta-analysis. One study was retrospectively conducted in an acute care hospital and the other two prospectively in LTCFs.

All participants in the studies were tested for SARS-CoV-2 by RT-PCR. In one of the studies (Stemler 2022),^23^ testing of participants (i.e. staff and visitors) was voluntary, and residents in the intervention LTCFs were not tested for SARS-CoV-2. Outcome data for the control facilities and the residents of the intervention facilities were obtained from the local health authority. This methodology likely introduced bias into the effect estimations.

The intervention showed a wide range of effects on the number of SARS-CoV-2 infections among patients undergoing index surgery, staff and residents of LTCFs from some reduction to an increase in the occurrence of infection. These resulted in substantial heterogeneity and as such we assessed the evidence as very low certainty for all outcomes reported.

The studies included in this review have serious bias issues, with our primary outcome measured differently. Hence, outcomes presented in a meta-analysis had substantial heterogeneity which may be because of methodological issues.

We are uncertain of the effect of asymptomatic testing in reducing SARS-CoV-2 transmission among staff and residents of LTCF, and patients undergoing index surgery because of very low certainty evidence, although the direction of the effect estimates favours asymptomatic testing group.^21,22^ The one study^23^ that showed there was no difference between asymptomatic testing compared to no routine testing in reducing SARS-CoV-2 infection only did voluntary (non-mandatory) testing of participants unlike the other two studies^21,22^ that showed an effect in favour of asymptomatic testing where testing of participants was mandatory. Asymptomatic testing may also reduce proportion of referrals in the intervention group compared to control.^21^ Put together, the certainty of the available evidence is very low. It is uncertain whether routine testing of asymptomatic people in healthcare facilities is effective in reducing transmission of SARS-CoV-2. One systematic review published in 2022 concluded that asymptomatic infections of HCWs varied greatly and with no strong evidence to reach any conclusion.^24^ Furthermore, public health experts have argued that prompt identification of asymptomatic COVID-19 cases will lead to early case isolation, which will minimise or stop the spread of SARS-CoV-2 to vulnerable individuals.^25^

## Conclusion

Our review provides insufficient evidence in support of asymptomatic testing of people for SARS-CoV-2 in healthcare settings to prevent transmission of the virus with overall very low certainty of the available evidence. There is a need for more and larger high quality RCTs in more diverse geographical settings to determine the effectiveness of asymptomatic testing of people for SARS-CoV-2 in healthcare facilities.

### Study limitations

The limitations of this systematic review are that only three observational studies having small sample size were included. Moreover, none of the studies were conducted in low-middle income countries. Therefore, the findings of this review cannot be generalised.
